# Video laryngoscopy in pre-hospital critical care – a quality improvement study

**DOI:** 10.1186/s13049-016-0276-6

**Published:** 2016-06-13

**Authors:** Marianne Grønnebæk Rhode, Mads Partridge Vandborg, Vibeke Bladt, Leif Rognås

**Affiliations:** Prehospital Critical Care Service, Aarhus University Hospital, Oluf Palmes Alle 32, 1, Aarhus, N, 8200 Denmark; Department of Pre-hospital Critical Care Services, The Central Denmark Region, Oluf Palmes Alle 34, Aarhus, N, 8200 Denmark; Pre-hospital Critical Care Service, Department of Anaesthesia, Viborg Regional Hospital, Heibergs Alle 4, Viborg, 8800 Denmark; Pre-hospital Critical Care Service, Department and Anaesthesia, Randers Regional Hospital, Skovlyvej 1, Randers, 8930 Denmark

**Keywords:** Pre-hospital, Prehospital, Out-of-hospital, Rapid sequence intubation, video laryngoscope, McGrath MAC, First-pass success rate, Difficult endotracheal intubation, Emergency medical services

## Abstract

**Background:**

Pre-hospital endotracheal intubation is challenging and repeated endotracheal intubation is associated with increased morbidity and mortality.

We investigated whether the introduction of the McGrath MAC video laryngoscope as the primary device for pre-hospital endotracheal intubation could improve first-pass success rate in our anaesthesiologist-staffed pre-hospital critical care services. We also investigated the incidence of failed pre-hospital endotracheal intubation, the use of airway adjuncts and back-up devices and problems encountered using the McGrath MAC video laryngoscope.

**Methods:**

Prospective quality improvement study collecting data from all adult pre-hospital endotracheal intubation performed by four anaesthesiologist-staffed pre-hospital critical care teams between December 15^th^ 2013 and December 15^th^ 2014.

**Results:**

We registered data from 273 consecutive patients. When using the McGrath MAC video laryngoscope the overall pre-hospital endotracheal intubation first-pass success rate was 80.8 %. Following rapid sequence intubation (RSI) it was 88.9 %. This was not significantly different from previously reported first-pass success rates in our system (*p* = 0.27 and *p* = 0.41). During the last nine months of the study period the overall first-pass success rate was 80.1 (*p* = 0.47) but the post-RSI first-pass success rate improved to 94.4 % (0.048).

The overall pre-hospital endotracheal intubation success rate with the McGrath MAC video laryngoscope was 98.9 % (*p* = 0.17). Gastric content, blood or secretion in the airway resulted in reduced vision when using the McGrath MAC video laryngoscope.

**Conclusion:**

In this study of video laryngoscope implementation in a Scandinavian anaesthesiologist-staffed pre-hospital critical care service, overall pre-hospital endotracheal first pass success rate did not change. The post-RSI first-pass success rate was significantly higher during the last nine months of our 12-month study compared with our results from before introducing McGrath MAC video laryngoscope. The implementation of the Standard Operating Procedure and check list for pre-hospital anaesthesia during the study period may have influenced the first-pass success rate and constitutes a potential confounder.

The potential limitations of the McGrath MAC video laryngoscope when there are gastric content, blood and secretions in the airways need to be further investigated before the McGrath MAC video laryngoscope can be recommended as the primary device in all pre-hospital endotracheal intubations.

**Electronic supplementary material:**

The online version of this article (doi:10.1186/s13049-016-0276-6) contains supplementary material, which is available to authorized users.

## Background

Advanced airway management is a vital but challenging skill in pre-hospital critical care and pre-hospital critical care providers must be competent in difficult airway management [1–3]. Limited access to the patient and certain biophysical conditions (e.g. obesity, short neck, face- and neck injuries, and anatomical restrictions) are predicting conditions for difficult airway management and difficult endotracheal intubation (DETI) and thus may complicate pre-hospital endotracheal intubation (PHETI) [1, 4, 5].

In both the template for uniform reporting data from pre-hospital advanced airway management by Sollid et al [6] and the “Practical guidelines for management of difficult airway” by the American Society of Anaesthesiologists [7], difficult endotracheal intubation is defined as more than one attempt needed to successfully perform endotracheal intubation.

PHETI first-pass success rates differ hugely between different pre-hospital emergency medical systems (EMS) and seem to be highly influenced by the organisation, staffing, case load and case mix of the EMS studied [2, 8, 9].

A study from our regional anaesthesiologist-staffed pre-hospital critical care system reported an overall PHETI first-past success of 77.6 % [10].

The incidence of failed PHETI in physician-staffed EMS is reported to be 1-2 % [9, 11, 12] which compares to the results from our own anaesthesiologist-staffed pre-hospital critical care service [10]. A metaanalysis by Lossius et al reported significantly increased PHETI success rates by physicians compared with non-physicians [11].

Complication rates seem to be correlated to repeated PHETI [9, 13] and both difficult and failed PHETI are associated with increased morbidity and mortality [1, 10, 14, 15]. In a study from an American emergency department with 1828 orotracheal intubations over a 4-year period, Sakles et al showed that the incidence of complications following orotracheal intubation increased from 14.2 % if the intubation was successful on the first attempt to 47.2 % if two attempts were needed [16] to secure the airway. Previous results from our own system showed an overall incidence of complications of 7.4 % in cases with first-pass success and 23.2 % when two endotracheal intubation (ETI) attempts were needed [10] to secure the airway. Following rapid sequence intubation (RSI), this difference was even more pronounced with complication rates rising from 11.4 % when first-pass success was achieved to 40.0 % when two PHETI attempts were needed [10].

In recent years, video laryngoscopy has been introduced to improve first-pass success rates and subsequent patient safety during in-hospital endotracheal intubation. Several in-hospital studies and case series have reported improved laryngeal views and greater ETI success rates compared with direct laryngoscopy [17–21] when using the video laryngoscope (VL) in patients with predicted or known difficult airways.

Video laryngoscopy has the potential to facilitate PHETI but the pre-hospital use of video laryngoscopy has not been widely examined and only a few reports exist on the use of VL to secure the airway in the pre-hospital setting [22–26]. Both studies performed on manikins simulating pre-hospital situations [27, 28] and on actual pre-hospital patients [23] report that video laryngoscopy seem to improve intubation conditions.

Following the previously mentioned PHETI-results from the anaesthesiologist-staffed pre-hospital critical care system in the Central Denmark Region [10], we gradually introduced the McGrath MAC video laryngoscope (Aircraft Medical Ltd., Edinburgh, UK) as the primary device for PHETI in this service.

We are not aware of any previous studies reporting airway management data following full-scale implementation of the McGrath MAC video laryngoscope as the primary device for PHETI in an anaesthesiologist-staffed pre-hospital critical care service.

The main purpose of the present study was to compare first-pass success rates after introduction of the McGrath MAC video laryngoscope as primary pre-hospital intubation device with the PHETI first-pass success rate previously reported from our system [10].

We also wanted to compare the incidence of failed PHETI and the use of airway adjuncts and back-up devices after introducing the McGrath MAC video laryngoscope with those previously reported from our system.

Thirdly, we aimed at investigating whether there were any problems in using the McGrath MAC in the pre-hospital setting.

Finally, we wanted to do subgroup analysis on data from patients who underwent pre-hospital RSI and patients in cardiac arrest.

## Methods

### Study design

We designed a prospective observational quality improvement study where we collected data from four anaesthesiologist-staffed pre-hospital critical care teams in accordance with the recommended template by Sollid et al [6].

### Setting

The Central Denmark Region covers a mixed urban and rural area of approximately 13000 km^2^ with a population of 1.270.000. The overall population density is 97.7 inhabitants pr. km^2^ (www.dst.dk/befolkning).

The region has a two-tiered EMS (Emergency Medical Service) system. The first tier consists of 64 ground ambulances staffed with Emergency Medical Technicians (EMTs) on an intermediate or paramedic level. EMTs in the Central Denmark Region do not perform ETI, nor do they use supraglottic airway devices (SADs).

The second tier consists of ten pre-hospital critical care teams comprising a consultant anaesthesiologist and an EMT. Nine of these teams are deploid by rapid response vehicles; the tenth team staffs a HEMS (Helicopter Emergency Medical Services) helicopter. The four pre-hospital critical care teams participating in this study employ approximately 50 consultant anaesthesiologists. Intensive care and critical care are core parts of the Danish specialist training in anaesthesiology.

None of the participating anaesthesiologists are full-time pre-hospital critical care physicians. All pre-hospital critical care physicians in our services deliver emergency anaesthesia and advanced airway management both in- and out-of-hospital on a regular basis.

All through the study period, all pre-hospital critical care teams carried the same equipment for airway management including: bag-valve-masks (BMV), endotracheal tubes and standard laryngoscopes with Macintosh blades, intubation stylets (14 Fr), *Airtrach*™ laryngoscopes (Prodol Meditec, Vizcaya, Spain), Gum-Elastic Bougies (14 Fr), standard single use Laryngeal Mask Airways (LMAs), Intubating LMA with endotracheal tube (FastTrach™, Teleflex Medical Europe, Westmeath, Ireland) and Quicktrach (VBM Medizintechnik GmbH, Sulz a. N., Germany) for establishing a surgical airway. For confirmation of correct laryngeal tube placement, all units used capnography, and they were all equipped with automated ventilators. All units carried a standardised set-up of medications.

In addition, the McGrath MAC video laryngoscope (Aircraft Medical Ltd., Edinburgh, UK) were gradually introduced as the primary choice for PHETI in the pre-hospital critical care teams. The McGrath MAC has a small camera and light source located at the distal end of the blade and a screen mounted on the handle. The single-use blade has standard Macintosh shape that allows direct laryngoscopy in addition to video laryngoscopy. During the study period, these blades were available in sizes 2, 3 and 4.

Prior to VL introduction, most anaesthesiologists employed in the involved pre-hospital critical teams had in-hospital experience in the use of VL. They also received a two hour long standardized training session with the McGrath MAC VL provided by the primary investigators. This session included a brief didactic introduction, a demonstration of the intubation technique and simulation training on manikins.

At the initiation of the study, the pre-hospital critical care service in our region had no standard operating procedures (SOPs) for PHETI or RSI and the anaesthesiologists used the available drugs at their own discretion. During the study period (starting 23^th^ of June, 2014), an SOP for pre-hospital anaesthesia and PHETI was introduced. This SOP includes a structured approach to pre-hospital advanced airway management and a pre-intubation check list and it recommends s-ketamine and suxamethonium as the drugs of choice for pre-hospital RSI (translated versions of the SOP and the check list are available as Additional files 1 and 2).

### Study period

Data collection took place from December 15^th^ 2013 to December 14^th^ 2014.

### Participants

Inclusion criteria: All adult patients in whom one of the four participating pre-hospital critical care teams attempted PHETI during the study period.

Exclusion criteria: Patients younger than 15 years.

### Variables

The pre-hospital critical care anaesthesiologists filled in a registration form containing core data as recommended by Sollid et al [6]. The registration form was largely the same as the one used in the previous study of PHETI in our system [10], but we included additional questions about the use of the McGrath MAC VL in the pre-hospital setting.

### Exposure variables

In our study, PHETI could be performed by using the VL, a standard laryngoscope (with standard Macintosh blades), the AirTraq ® laryngoscope or though the ILMA. PHETI could be performed with or without a standard intubation stylet and with or without the use of a gum-elastic bougie. Other airway management techniques available were BVM-ventilation, using an LMA/ILMA or establishing a surgical airway. We registered the primary device used to secure a patent airway and the use of any back-up device.

PHETI could be performed without the assistance of drugs, as drug-assisted PHETI or as RSI. We defined drug-assisted PHETI as PHETI performed with any combination of analgesic or sedative drugs without the use of a Neuro Muscular Blocking Agent (NMBA) and RSI as the use of any combination of a sedative or an analgesic drug and a NMBA.

### Endpoints

#### The primary endpoint was

The overall first-pass success rate, defined as only one attempt needed to successfully performed PHETI.

#### Secondary endpoints were

- First-pass success rate following RSI and in patients in cardiac arrest (CA).
- The incidence of failed PHETI. We defined failed PHETI as cases where PHETI was impossible in the pre-hospital setting.
- Airway back-up devices used.
- The anaesthesiologists’ reasons for using methods other than the McGrath MAC VL as the primary device for PHETI. The reasons were categorised as: i) being to unfamiliar with the McGrath MAC, ii) estimating it would take too long time to use the McGrath MAC VL as compared with using the conventional Macintosh laryngoscope, iii) the McGrath MAC VL was not available or iv) other reasons.
- Problems encountered when using the McGrath MAC VL in the pre-hospital setting categorised as: i) difficulties in introducing the VL or inserting the endotracheal tube, ii) poor overview (e.g. because of difficult anatomy, suboptimal positioning of the patient, airway bleeding or airway secretions), iii) technical problems (poor screen vision or troublesome light conditions) or iv) other problems.

To reduce the potential effects of the physicians’ VL learning curve on the results we did a separate analysis of the data collected during the last 9 months of the 12 months study period.

### Ensuring data quality

To ensure the highest possible data coverage the registration forms were continuously cross-checked with the standard pre-hospital records by the primary investigators. If a registration form was missing, the attending anaesthesiologist was contacted and asked to fill in the registration form retrospectively.

### Study size

Based on the literature, we estimated that the overall first-pass success rate may be improved from approximately 78 % [10] to 88 % by introducing the McGrath MAC VL in the pre-hospital critical care teams.

Sample size calculations made in the statistical program Stata 13 (StataCorpLP) showed that it would require 209 patients in which the McGrath MAC VL were used as the primary device for pre-hospital intubation to detect a difference of this magnitude with 90 % power at a significance level of 5 %. Based on these calculations, we estimated that we would need to collect data for one year from the four participating pre-hospital critical care teams.

### Statistics

The data was analysed in the statistical program Stata 13 (StataCorpLP). We compared the results from the current study with the results from our system prior to the implementation of the McGrath MAC VL using the chi-squared test except when data was scarce, in which case we applied the Fisher’s exact test to test the hypothesis of no association.

We consider a *p*-value below 0.05 as being statistically significant.

Missing data were rare. If we could not obtain the missing data, we performed complete case analyses.

### Ethics

Because of its quality improvement nature, The Regional Medical Ethics Committee confirmed that the project did not need their approval.

The Danish Data Protection Agency approved the study (Journal number 2013-41-1462).

## Results

During the 12-months study period the involved pre-hospital critical care teams attempted endotracheal intubation in 273 patients. In 93.8 % (*n* = 256) the anaesthesiologists chose the McGrath MAC VL as the primary pre-hospital endotracheal intubation device.

### Pre-hospital endotracheal intubation first-pass success rates

The overall first-pass success rate for the entire 12-month study period was 80.8 % (*n* = 207). This is not significantly different (*p* = 0.27) from the 77.6 % pre-VL first-pass success rate in our system [10].

During these 12 months, RSI was performed in 42.2 % (*n* = 108) of the patients and with a first-pass success rate of 88.9 %, (*n* = 96) (*p* = 0.41 compared to pre-VL results). In cardiac arrest patients the first-pass success rate was 76.8 % (*n* = 174).

Analysis of the last nine months of the study period showed an overall first-pass success rate of 80.1 % (*n* = 176) (*p* = 0.47 when compared with pre-VL data), while the post-RSI first-pass success rate significantly improved to 94.4 % (*n* = 71) (*p* = 0.048 when compared with pre-VL data). For patients in cardiac arrest the first-pass success rate in this period was 74.6 % (*n* = 113).

### Incidence of failed pre-hospital endotracheal intubation

Using the McGrath MAC VL, the overall success rate was 98.9 % (*n* = 253) (*p* = 0.13 when comparing with the 99.7 % in the pre-VL material). Of the 3 cases of failed intubations, one was a case of facial trauma managed by BVM-ventilation. The patient had a surgical airway established in the emergency room. The two last patients were patients in cardiac arrest managed by uneventful BVM-ventilation.

### Airway adjuncts and back-up devices used

In 96.1 % (*n* = 246) of the cases the anaesthesiologists used a stylet and in 0.8 % of the cases (*n* = 2) a Gum-elastic Bougie were used to guide the endotracheal tube.

### Problems encountered when using the McGrath MAC video laryngoscope

The physicians participating in our study reported that the McGrath MAC VL had limitations if there was gastric content, blood or secretion in the airway. In these situations, it was as if there was a film covering the light source resulting in impaired luminosity and vision.

### Reasons for not using the McGrath MAC video laryngoscope

The most common reason for not using the McGrath MAC VL as primary device for PHETI (*n* = 17), was expected poor visualisation due to either blood, water or gastric contents in the airway (2.6 %, *n* = 7) or anticipated sunlight on the screen (1.1 %, *n* = 3)

## Discussion

### Pre-hospital endotracheal intubation first-pass success rates

Overall pre-hospital endotracheal first pass success rate did not change after the introduction of the McGrath MAC video laryngoscope as the primary intubation device. The post-RSI first-pass success rate was higher during the last nine months of our study compared with the previous results from our system and this met statistical significance (*p* = 0.048). This could indicate, that introducing the McGrath MAC as the primary device for PHETI may improve patient safety during pre-hospital RSI. The sample size of the last nine months does not allow us to conclude on the negative results of the comparison of overall first-pass success rates between the last nine months and the pre-VL data. This does not apply to the positive finding of a significant improvement in post-RSI first-pass success rate during the last nine months compared with our pre-VL data.

A post-RSI first-pass success rate during the last nine months of the study at 94.4 % compares to the best international results [12, 29].

Although other definitions of difficult PHETI than the one suggested by Sollid et al [6] and used in this paper are sometimes used in the pre-hospital literature, an overall first-pass success rate of 80.8 % in this study is rather low compared with other anaesthesiologist-staffed pre-hospital critical care systems [9, 12, 30]. A newly published multicentre study collected data from physician-staffed helicopter emergency services in 6 countries [9] reported a first-pass success rate in PHETI of 85.5 %. A retrospective analysis from a physician-based EMS in Zurich, Switzerland found that the first pass success rate was as high as 96.8 % [12]. The reason for this difference is unknown but in our study cardiac arrest patients showed significantly lower first-pass success rates compared with RSI-patients. Aspiration is a frequent complication to cardiac arrest [31–33] and according to the participating physicians in our study it seems as if the McGrath Mac VL has limitations when there is gastric content, blood or secretion in the airway. Moreover, to minimise “hands-off time” during cardiac arrest, PHETI is often performed during on-going chest compression and with the patient lying on the ground. This may make PHETI in cardiac arrest patients more challenging compared with non-cardiac arrest patients. Variations in case mix may therefore have contributed to the observed differences in overall first-pass success rates in different systems.

The difference between the post RSI first-pass success rate calculated for the entire study period and the post-RSI first pass success rate during the last nine months may indicate that in-hospital training and experience may not always be sufficient even when using the same equipment as in the in-hospital setting.

We cannot rule out the possibility that the new SOP and check list for PHETI and RSI may have influenced our results. The SOP and check list were introduced during the study period to ensure a uniform approach to pre-hospital anaesthesia and thereby minimizing the risk of unexpected incidents and complications. Greater focus on optimizing the position of the patient and the airway assessment before PHETI may have improved the first-pass success rate by itself.

### Airway adjuncts and back-up devices used

Frequent use of an intubation stylet or Gum-elastic Bougie together with a VL corresponds to what has previous been reported [19].

### Incidence of failed pre-hospital endotracheal intubation

The 1.1 % incidence of failed PHETI compares to those reported from other physician-staffed EMS/HEMS in Europe [9, 11, 12, 29].

### Problems encountered when using the McGrath MAC video laryngoscope and reasons for not using the McGrath MAC video laryngoscope

There are few other studies reporting data on this. In a newly published study from Japan, intubation was performed using the McGrath Mac VL in a simulated hematemesis and vomitus setting. No difficulties with visualisation on the screen were described and success rate was comparable with direct laryngoscopy [34]. The reason for this apparent difference is not known.

Reflection of the sunlight on the screen is described as a potential problem but may be managed by attaching a reflection prevention filter over the screen or pulling a blanket/jacket over the intubating anaesthesiologists [35].

### Limitations

Data recording was done by the treating physicians, making our results susceptible to registration- and recall bias.

Using the Utstein-style template with nearly 50 variables to be registered for each patient there may also be a risk of registration fatigue, errors and missing data, even though most of the physicians were familiar with the template from previous studies.

The implementation of a novel SOP for PHETI during the study period may have influenced the results of this study and constitutes a potential confounder.

### Generalisability

In this prospective quality improvement study, data was collected from a homogenous Danish anaesthesiologist-staffed critical care system. The generalisation to other pre-hospital systems with different staffing, case mix and case load may be debatable. Still, we believe that our results may be transposed to other physician-staffed pre-hospital critical care services.

### Perspectives

Our results may have impact on the standard operating procedures for pre-hospital endotracheal intubations in pre-hospital critical care services with staffing, case load and case mix comparable to those in our service.

## Conclusion

In this study of video laryngoscope implementation in a Scandinavian anaesthesiologist-staffed pre-hospital critical care service, overall pre-hospital endotracheal first pass success rate did not change following the introduction of the McGrath MAC video laryngoscope as the primary intubation device. Post-RSI first-pass success rate was significantly higher during the last nine months of our study compared with our results from before introducing McGrath MAC video laryngoscope. This may indicate, that introducing the McGrath MAC as the primary device for pre-hospital endotracheal intubations could improve patient safety during pre-hospital RSI. The implementation of the Standard Operating Procedure and check list for pre-hospital anaesthesia during the study period may also have influenced the first-pass success rate and constitutes a potential confounder.

The potential limitations of the McGrath MAC video laryngoscope when there are gastric content, blood and secretions in the airways need to be further investigated before the McGrath MAC video laryngoscope can be recommended as the primary device in all pre-hospital endotracheal intubations.

Additional studies are necessary to further explore the effects of introducing the McGrath MAC in anaesthesiologist-staffed pre-hospital critical care.

## Abbreviations

BMV, Bag-valve-mask; CA, cardiac arrest; DETI, Difficult endotracheal intubation; EMS, Emergency medical systems; EMT, Emergency Medical Technicians; ETI, Endotracheal intubation; HEMS, Helicopter emergency medical service; ILMA, Intubating laryngeal mask; LMA, Laryngeal mask; NMBA, Neuromuscular blocking agent; PHETI, Pre-hospital endotracheal intubation; RSI, Rapid sequence intubation; SAD, Supraglottic airway devices; SOP, Standard operating procedures; VL, Video laryngoscope.

## Additional files

Additional file 1: Standard Operating Procedure for Pre-hospital anaesthesia. (DOC 33 kb) Additional file 2: Check list for pre-hospital anaesthesia and endotracheal intubation. (DOC 45 kb)
