# Supplementary material for: Video laryngoscopy in pre-hospital critical care – a quality improvement study
Source: Scand J Trauma Resusc Emerg Med. 2016 Jun 13;24:84. doi: 10.1186/s13049-016-0276-6 (PMC4906985; doi:10.1186/s13049-016-0276-6)
Supplement: Additional file 1: — Standard Operating Procedure for Pre-hospital anaesthesia. (DOC 33 kb) [file 13049_2016_276_MOESM1_ESM.doc]

Additional File 1:

Standard Operating Procedure for Pre-hospital anaesthesia.

Recommendations for pre-hospital Critical Care physicians in the Central Denmark Region.

**Purpose:**

- To ensure first-pass success during pre-hospital endotracheal intubation
- To avoid desaturation (SpO2 >92%) during pre-hospital endotracheal intubation
- To avoid hypotension (systolic blood pressure < 90 mmHg, < 120 mmHg in patients with a severe intracerebral event / head trauma) during pre-hospital endotracheal intubation.

**Preparations:**

- Optimize the setting, consider moving the patient before inducing anaesthesia
- Optimize the position of the patient, consider reverse Trendelenburg position if the patient is hemodynamically stable.
- Preoxygenation: Non-rebreather reservoir oxygen mask at 15 lpm for at least 3 minutes or bag-valve-mask with high-flow oxygen if the patients ventilation is insufficient
- Airway Assessment.
- Consider using McGrath MAC video laryngoscope (e.g. AirTrach), Gum-elastic-bougie and other alternatives.
- In high risk patients; consider
- Bag-valve-mask ventilation before endotracheal intubation, if SpO2 < 92%
- Nasal cannula with high flow oxygen through the apnoeic period
- Prepare and communicate a ”Plan A, B and C”
- Go through the check list for pre-hospital anaesthesia and intubation
- If the patient has been fittred with a cervical collar, remove it and do manual in-line immobilization of the head and neck during laryngoscopy

**Standard procedure for induction of general anaesthesia:**

**Children < 6 months:**

- I.v. **S-ketamine 1mg/kg**
- I.v.. **Succinylcholine 2mg/kg**
- Consider i.v. Atropine 0.02 mg/kg
- Careful bag-mask ventilation between administration of medication and laryngoscopy

**Children > 6 months:**

- I.v. **S-Ketamine 1mg/kg**
- I.v. **Succinylcholine 2 mg/kg**
- Careful bag-mask ventilation between administration of medication and laryngoscopy

**Adults:**

- Consider i.v. **alfentanil** if the patient is hemodynamically stable
- I.v. **S-ketamin 1 mg/kg i.v**
- I.v. **Succinylcholine 1mg/kg -**  Consider i.v. Rocuronium (1mg/kg)

Tracheal placement of the tube **must** be confirmed by auscultation as well as with detection of end-tidal cabon dioxide (EtCO_2_).

**Standard maintance of anaesthesia:**

- I.v. **Fentanyl** in combination with i.v. **Midazolam**
- Alternatively propofol infusion or S-ketamin bolus (0.2-0.3 mg/kg)
- Consider i.v. **Rocuronium**

**Controlled ventilation:**

- If possible, pre-hospital controlled ventilation should be provided by using an **automated ventilator.**
- Ensure normoventilation – end-tidal CO_2_ must be continuously monitored.
