# Supplementary material for: Video laryngoscopy in pre-hospital critical care – a quality improvement study
Source: Scand J Trauma Resusc Emerg Med. 2016 Jun 13;24:84. doi: 10.1186/s13049-016-0276-6 (PMC4906985; doi:10.1186/s13049-016-0276-6)
Supplement: Additional file 2: — Check list for pre-hospital anaesthesia and endotracheal intubation. (DOC 45 kb) [file 13049_2016_276_MOESM2_ESM.doc]

Additional File 2

Check list for pre-hospital anaesthesia and endotracheal intubation

If immediately endotracheal intubation is of the essence, only go through the red points in the check list.

| Patient accept |  | Check |
| --- | --- | --- |
| Setting and patient position | Optimized |  |
| Airway assesment | Done |  |
| Oxygen supply | Functioning, back-up available |  |
| Preoxygenation | Reservoirmask/bag-valve-mask ventilation, 3 minutes |  |
| Monitoring equipment | Pulse, blood pressure and SpO_2_ |  |
| i.v. /i.o. access | Consider extra access |  |
| Suction | Available and functioning |  |
| BVM-equipment | Bag-valve-mask, reservoir, oxygen tube |  |
| Endotracheal tube | Chosen size, with stylet |  |
| Syringe for cuff inflation | 10 ml |  |
| Laryngoscope | Chosen type, functioning |  |
| Laryngoscope blade | Correct size |  |
| Capnograph | Present and functioning |  |
| Tube fixation equipment | Present |  |
| Ventilator | Ready |  |
| S-ketamine 100 mg | In a 10 ml syringe |  |
| Suxamethonium 100 mg | In a 5 ml syringe |  |
| (Alfentanil) | (In a 2 ml or 10 ml syringe) |  |
| Maintance of anesthesia | According to SOP |  |
| Distribution of roles | E.g medication, in-line stabilization |  |
| Plan A, B and C | Planned and communicated to the entire team |  |
| Back-up devices according to Plan B and C | Available |  |
